# Detecting Functional Impairment Among Adolescents in South Africa Using Culturally Adapted Assessments

**DOI:** 10.1192/j.eurpsy.2023.221

**Published:** 2023-07-19

**Authors:** K. Zarei, A. Lundin, L. Carvajal Velez

**Affiliations:** 1 NIMHD, Bethesda, United States; 2 Global Public Health, Karolinska Institutet, Stockholm, Sweden; 3 Division of Data, Analytics, Planning and Monitoring, Data and Analytics Section, UNICEF, New York, United States

## Abstract

**Introduction:**

Functional impairment (FX) screening tools could potentially be used in resource-limited settings to identify adolescents who need mental health support.

**Objectives:**

Culturally adapted, isiXhosa versions of FX questions and the Patient Health Questionnaire-9 (PHQ-9) and the Generalized Anxiety Disorder-7 (GAD-7) were used to assess depression (MDD) and anxiety (GAD) among adolescents (10-19 years) in South Africa.

**Methods:**

Adolescents were recruited from the general population and from nongovernmental organizations working with those in need of mental health support. The PHQ-9 and GAD-7 were previously culturally adapted, translated into isiXhosa, and administered to 302 adolescents (10-19 years old, 56.9% female), and three culturally adapted items were asked to assess functional impairment regarding problems that 1) interfere with activities/relationships at home, 2) interfere with activities at school/work, and 3) cause any issues with peers. FX items were dichotomized into at least some impairment (“sometimes” and “often”) and no impairment (“rarely” and “never”). The Kiddie Schedule for Affective Disorders and Schizophrenia (K-SADS) was administered by trained clinicians as the gold standard measures for MDD, GAD, and FX. To assess criterion validity against a clinician’s diagnosis, we used total PHQ-9 and GAD-7 scores as well as combined FX and PHQ-9 and GAD-7 scores to construct receiver operating characteristic curves, and calculated the area under the curve (AUC) for each test as well as other psychometric properties.

**Results:**

In the sample, 32.1% and 17.9% of adolescents screened positive for moderate to severe MDD and GAD respectively with the culturally adapted PHQ-9 and GAD-7. Among adolescents, 39.7%, 37.1%, and 29.1% reported at least some impairment at home, school, and among peers respectively. Spearman correlations between the three items (Cronbach’s Alpha = 0.69) ranged from 0.35-0.53, and kappa statistics ranged from 0.18-0.47. For the culturally adapted PHQ-9, the AUC was 0.86 for the full sample. A score of >=10 had 97% sensitivity and 75% specificity for detecting MDD. For the culturally adapted GAD-7, the area under the curve was 0.69, and cutoff scores with an optimal sensitivity-specificity balance were low (>=6) and had 76% sensitivity and 69% specificity for detecting GAD. For the combination of the culturally adapted PHQ-9 with the FX questions, the AUC was 0.80 for the sample, and a score of >=10 had 77% sensitivity and 83% specificity for detecting adolescents with MDD. For the combination of the culturally adapted GAD-7 with the FX questions, the AUC was 0.68, and a score >=6 had 70% sensitivity and 76% specificity for detecting adolescents with GAD.

**Conclusions:**

While the culturally adapted FX questions didn’t enhance the assessment of MDD and GAD among adolescents in South Africa, these items still provide an opportunity to measure FX in different settings.

**Disclosure of Interest:**

None Declared

